# The Extracellular Domain of Myelin Oligodendrocyte Glycoprotein Elicits Atypical Experimental Autoimmune Encephalomyelitis in Rat and *Macaque* Species

**DOI:** 10.1371/journal.pone.0110048

**Published:** 2014-10-10

**Authors:** Alan D. Curtis, Najla Taslim, Shaun P. Reece, Elena Grebenciucova, Richard H. Ray, Matthew D. Rosenbaum, Robert L. Wardle, Michael R. Van Scott, Mark D. Mannie

**Affiliations:** 1 Department of Microbiology and Immunology, East Carolina University Brody School of Medicine, Greenville, NC, United States of America; 2 The Department of Physiology, East Carolina University Brody School of Medicine, Greenville, NC, United States of America; 3 The Department of Comparative Medicine, East Carolina University Brody School of Medicine, Greenville, NC, United States of America; 4 The Harriet and John Wooten Laboratory for Alzheimer’s and Neurodegenerative Disease Research, East Carolina University Brody School of Medicine, Greenville, NC, United States of America; 5 The University of Chicago, Department of Neurology, Chicago, IL, United States of America; Hospital Nacional de Parapléjicos - SESCAM, Spain

## Abstract

Atypical models of experimental autoimmune encephalomyelitis (EAE) are advantageous in that the heterogeneity of clinical signs appears more reflective of those in multiple sclerosis (MS). Conversely, models of classical EAE feature stereotypic progression of an ascending flaccid paralysis that is not a characteristic of MS. The study of atypical EAE however has been limited due to the relative lack of suitable models that feature reliable disease incidence and severity, excepting mice deficient in gamma-interferon signaling pathways. In this study, atypical EAE was induced in Lewis rats, and a related approach was effective for induction of an unusual neurologic syndrome in a cynomolgus macaque. Lewis rats were immunized with the rat immunoglobulin variable (IgV)-related extracellular domain of myelin oligodendrocyte glycoprotein (IgV-MOG) in complete Freund’s adjuvant (CFA) followed by one or more injections of rat IgV-MOG in incomplete Freund’s adjuvant (IFA). The resulting disease was marked by torticollis, unilateral rigid paralysis, forelimb weakness, and high titers of anti-MOG antibody against conformational epitopes of MOG, as well as other signs of atypical EAE. A similar strategy elicited a distinct atypical form of EAE in a cynomolgus macaque. By day 36 in the monkey, titers of IgG against conformational epitopes of extracellular MOG were evident, and on day 201, the macaque had an abrupt onset of an unusual form of EAE that included a pronounced arousal-dependent, transient myotonia. The disease persisted for 6–7 weeks and was marked by a gradual, consistent improvement and an eventual full recovery without recurrence. These data indicate that one or more boosters of IgV-MOG in IFA represent a key variable for induction of atypical or unusual forms of EAE in rat and *Macaca* species. These studies also reveal a close correlation between humoral immunity against conformational epitopes of MOG, extended confluent demyelinating plaques in spinal cord and brainstem, and atypical disease induction.

## Introduction

Multiple sclerosis (MS) is an inflammatory demyelinating disease of the central nervous system (CNS) characterized by focal inflammatory lesions and demyelinating plaques in periventricular and perivascular regions of the CNS [1]–[3]. MS is a clinically heterogeneous disease with substantial variability in both clinical presentation and disease progression. Experimental autoimmune encephalomyelitis (EAE) is a common model of MS used to investigate immunopathogenesis and test potential therapeutics [4]–[6]. Research in EAE has focused on ‘classical’ models in which disease progression is marked by a stereotypical ascending flaccid paralysis. Rats afflicted with classical EAE initially present with flaccid paralysis of the distal tail followed by an anterior progression over the next 1–3 days culminating in a symmetric paralysis of both hindlimbs. Classical disease courses are monophasic, relapsing-remitting, or chronic-progressive depending on the rodent strain and immunizing antigen, with focal mononuclear inflammatory lesions observed in the spinal cord and brainstem. However, a stereotypical ascending flaccid paralysis, which is the defining clinical hallmark of classical EAE, is seldom observed in MS.

Variants of EAE that feature ‘atypical’ clinical signs may be more representative of disease heterogeneity in MS. Atypical forms of EAE are marked by a lack of ascending paralysis, irregular disease progression, and substantial inter-animal heterogeneity in onset and presentation of clinical signs. Atypical EAE in mice and rats is often marked by: (a) axial-rotary torticollis/head-tilt (vertigo), (b) rigid or spastic asymmetric paralysis or unilateral forelimb and/or hindlimb weakness, (c) unilateral ataxia, (d) paralysis without tail involvement, (e) uneven or sporadic disease progression, and (f) in some models by a delayed onset and a prolonged non-resolving time course [7]–[11]. The torticollis often progresses to the extent that the rodent will continually rotate about the body axis, presumably in an effort to right itself, possibly reflecting a sensory deficit of the vestibular control centers. Unlike classical forms of EAE, several studies have noted that atypical disease features an abundance of inflammatory lesions in the brain including the midbrain, cortex, and cerebellum.

Although atypical EAE may more closely reflect the diverse pathogenic processes of MS, the focus on classical EAE rather than atypical EAE is primarily due to a lack of models with consistent clinical signs of atypical disease in wildtype animals. The most robust models of atypical EAE are in mouse strains genetically deficient in the interferon gamma (IFN-γ) signaling pathway [12] but these mice have abnormal immune systems that cannot be used to accurately model MS. Atypical EAE in susceptible wildtype mouse strains has a variable incidence. Some mice initially develop atypical EAE but spontaneously transition to an ascending flaccid paralysis, whereas other mice of the same cohort solely develop classical disease [8]–[10]. Thus, there is a need for atypical models of EAE that are inducible and feature reliable and uniform incidence of severe atypical disease.

EAE in the Lewis rat is a widely used model for MS. Lewis rats given a single immunization with myelin basic protein in complete Freund’s adjuvant (CFA) exhibit a classical monophasic course of EAE marked by 3–5 days of tail and hind-limb paralysis followed by complete spontaneous remission [13]. Immunization of Lewis rats with myelin oligodendrocyte glycoprotein (MOG)-derived peptides or proteins in CFA causes a mild monophasic form of EAE [14]. MOG is present on the outer-surface of CNS myelin and is a quantitatively minor protein component of the myelin sheath. MOG is a target for demyelinating antibody (Ab) in humans and non-human primates (NHP) and causes EAE in NHP [15]–[22]. MOG also causes EAE in mice in spontaneous and induced disease models by Ab-dependent and independent mechanisms [22]–[25]. In contrast, NHP often exhibit a hyperacute neurologic disease marked by hemorrhagic/necrotic lesions in the brain that resembles fulminant forms of MS [18]–[21]. Overall, development of suitable EAE models in NHP has been problematic due to excessive susceptibility of NHP to EAE, aggressive severe disease, rapid progression to humane endpoints, neutrophilic and/or hemorrhagic inflammatory CNS lesions, and an insufficient time window for therapeutic intervention.

An atypical model of EAE in the Lewis rat and NHP would represent an important advance because such a model would enable vetting of novel therapeutic interventions that may have more direct relevance for drug development in MS. Herein, we show that Lewis rats immunized with the immunoglobulin variable (IgV)-related extracellular domain of MOG (IgV-MOG) in CFA and subsequently boosted with IgV-MOG in incomplete Freund’s adjuvant (IFA) exhibited high incidence of a severe, chronic course of atypical EAE. A similar immunization/boost strategy via repeated administration of IgV-MOG in IFA also induced a self-limited bout of nonclassical EAE in a cynomolgus macaque. This study thereby introduces a unique protocol to induce atypical/non-classical EAE as an advancement in model development for MS.

## Materials and Methods

### Ethics statement

Lewis rats (*Rattus norvegicus*) and the cynomolgus macaque (*Macaca fascicularis*) were housed at East Carolina University. Animal care and use were performed in accordance with approved animal use protocols for Lewis rats (K144b), the cynomolgus macaque (K154), and institutional guidelines of the East Carolina University’s Institutional Animal Care and Use Committee. All injections were given to rats and the cynomolgus macaque while under isoflurane anesthesia (Abbott Laboratories, Chicago, IL). During expression of EAE, rats were monitored twice per day, and the NHP was monitored three times per day.

A colony of cynomolgus macaques (*Macaca fascicularis*) born between 1998 and 2006 was maintained at East Carolina University. Twice annually, the colony was monitored for simian retroviruses, simian T-cell leukemia virus, herpes B virus, and simian immunodeficiency virus via multiplexed fluorometric immunoassays. In addition, macaques were tuberculin-tested intradermally. Annually, the colony underwent fecal analysis for parasites and fecal culture for *Shigella*, *Campylobacter*, and *Salmonella* species. The macaques had *ad libitum* access to water via an automated watering device, were fed chow twice daily (LabDiet Monkey Diet 5038, Purina Laboratory, St Louis, MO), and were supplemented daily with fruits and vegetables. The light cycle was maintained on a 12∶12-hour light:dark cycle. Macaques were housed indoors in same-sex groups of 2 to 6 macaques in stainless steel caging that included gang-caging and vertical tunnels connecting upper and lower batteries to provide vertical mobility and voluntary visual and physical separation from cagemates. Temporary separation of macaques for experimental monitoring or clinical interventions was accomplished by using screens that maintained visual and tactile connection whenever feasible. The fresh-air supply to rooms housing macaques was filtered at MERV 8 (<3 to 10 µm particle diameter) to minimize exposure to environmental allergens. The macaques were assigned to IACUC-approved protocols and were maintained in accordance with the USDA Animal Welfare Act and regulations and the *Guide for the Care and Use of Laboratory Animals*. The animal care and use program at East Carolina University is USDA-registered, maintains a Public Health Services Assurance, and is fully accredited by AAALAC.

The subject in this study was initially housed with a cage-mate. Daily enrichments consisted of various items within the enclosure (ropes, tennis balls, stuffed toys, etc.), radio music, and a variety of fresh fruits, vegetables, seeds, and cereals for snacking. Other enrichment included access to a water tub for swimming activities and television and/or movies. All antigen injections were in IFA and were given to the cynomolgus macaque while under isoflurane anesthesia. The subject was directly observed once per day until disease onset and then at least three times per day in 30 minute sessions during overt disease. In addition, a web camera was placed in the room for remote monitoring at any time by the veterinary staff and investigators. Upon onset of EAE, the subject was housed separately in caging that included at least 4 feet of vertical height and 8 square feet of floor space. The subject had voluntary visual contact with other animals within the room. Hydration was supplemented with fluids such as Gatorade or Kool-Aid. Fruit and vegetable enrichments were given at least twice per diem. All measures were taken to ensure the safety and well-being of the subject. After 730 days of observation and 482 days after spontaneous recovery from EAE, the subject was euthanized with intravenous sodium pentobarbital (100 mg/kg) following anesthesia with intramuscular Telazol (4 mg/kg). Tissues were collected and analyzed following a complete necropsy.

### Expression systems for Full Length MOG and IgV-MOG

RNA was purified from rat or macaca CNS tissue by use of the TRizol Reagent (Life Technologies, Carlsbad, CA). Four expression vectors were constructed for this study. Rat and macaca cDNA encoding the IgV domain of MOG and full length MOG protein were amplified from total CNS RNA by RT-PCR. Rat and macaca DNA species encoding the IgV-MOG domain were inserted into the pQE-40 prokaryotic expression vector (Qiagen, Valencia, CA), whereas the rat and macaca DNA sequences encoding the full length proteins were inserted into the pIRES2-AcGFP1 mammalian expression vector (Clontech, Mountain View, CA). Directional restriction site-free overlap extension was used to insert the respective DNA into the expression vectors.

The pQE-40 recombinant vectors were used to express rat and macaca IgV-MOG as inclusion bodies in the M15[pREP4] strain of *E. coli*. The N-terminus of the protein (MRGSHHHHHHGSGI) was encoded by the vector. The adjoining sequence represented the rat or macaca 1–123 amino acid sequence of IgV-MOG that replaced a vector-encoded murine dihydrofolate reductase sequence. The IgV-MOG sequence did not include the signal sequence and spanned amino acid positions from GQFRVIGP……VEDPFYWIN for the rat protein and GQFRVIGP……VEDPFYWVS for the macaca protein. The 6xhistidine N-terminal sequence was used as an affinity tag for purification of the protein.

The recombinant pIRES2-AcGFP1 expression vectors were engineered to encode full length rat and macaca MOG as transmembrane surface proteins. The full length proteins included the native MOG signal sequences to direct export through the Golgi apparatus to the cell surface so that the recombinant proteins would have the predicted patterns of N-linked glycosylation. These expression vectors were transfected into 293F human embryonic kidney cells (HEK) by use of TurboFect transfection reagent (Thermo Fisher Scientific, Waltham, MA). Stable lines were obtained by drug selection and use of a Becton Dickinson FACSVantage cell sorter (Franklin Lakes, NJ) to isolate cells that expressed high levels of green fluorescent protein (GFP) and cell surface MOG.

### Expression and purification of IgV-MOG

Recombinant rat and macaca IgV-MOG proteins were purified from transformed *E. coli* M15[pREP4] cells after 3–4 hours of induction with isopropyl β-D-1-thiogalactopyranoside. Cell pellets were lysed in 8 M urea (with 100 mM NaH_2_PO_4_, 10 mM Tris-Cl, pH 8.0). Proteins were purified by affinity chromatography on Ni-NTA agarose columns (Qiagen, Valencia, CA). After elution, proteins were refolded at 500 µg/ml by stepwise dialysis in buffer A (4 M urea, 50 mM glycine, 15.6 mM NaOH, 10% w/v sucrose, 1 mM EDTA, 1 mM reduced glutathione, 100 µM oxidized glutathione at pH 9.6), buffer B (60 mM ethanolamine, 10% w/v sucrose, 1 mM EDTA, 100 µM reduced glutathione, and 10 µM oxidized glutathione at pH 9.6), and finally in 20 mM sodium acetate buffer (pH 4.0). Alternatively, protein preparations were reduced prior to folding via incubation with 20 mM TCEP-HCl (EMD, Billerica, MA) and dialyzed against 4 M urea (with 100 mM NaH_2_PO_4_, 10 mM Tris-Cl, pH 8.0) to remove the TCEP-HCl. Proteins were then refolded at approximately 500 µg/ml by stepwise dialysis against buffer A, buffer B, and ultrapure ‘Milli-Q’ H_2_O. Soluble preparations were concentrated in Amicon Ultra-15 centrifugal filter devices (MilliPore, Billarica, MA). Protein quantity was assessed by absorbance at 280 nm and purity was assessed by SDS-PAGE. Protein preparations were aliquoted and stored frozen at −80°C.

### Induction and measurement of EAE in rat and Macaca species

A synthetic peptide representing the dominant encephalitogenic sequence of guinea pig myelin basic protein (GP69-88) (Y-G-S-L-P-Q-K-S-Q-R-S-Q-D-E-N-P-V-V-H-F) was purchased from Quality Controlled Biologicals, Inc. (Hopkinton, MA). To elicit EAE, Lewis rats were injected with an emulsion containing designated doses of GP69-88 and/or rat IgV-MOG in CFA. The CFA emulsion was prepared by mixing 1 part antigen in buffered saline and 1 part CFA (4 mg/ml of heat killed *Mycobacterium tuberculosis* H37Ra, Difco, Franklin Lakes, NJ per 1 ml of IFA) followed by sonication on ice until the emulsion was thick, viscous, and stable in water. The emulsion contained a final dose of 200 µg of heat killed *Mycobacterium tuberculosis* in 0.1 ml volume which was injected as two 0.05 ml subcutaneous injections on either side of the base of the tail for each rat. Where indicated, subsequent booster immunizations consisted of 200 µg rat IgV-MOG in IFA. All immunized rats were weighed and scored daily for classical or atypical signs of EAE and were observed twice daily during active disease. The following scale was used to score *classical EAE*: no clinical disease = 0; paralysis in the distal tail = 0.25; limp tail = 0.5; ataxia = 1.0; hindlimb paresis = 2.0; full hindlimb paralysis = 3.0. Ataxia was scored as an uneven or wobbly gait. Hind leg paresis was scored as the retention of some voluntary ambulatory movement in the hind limbs but without the ability to ambulate upright. Rats exhibiting *atypical EAE* were scored by the following scale: no overt clinical signs = 0; forelimb weakness without hindlimb involvement = 0.25; ataxia without flaccid paralysis of the tail = 0.5; rigid asymmetric extension of a hindlimb or forelimb (as opposed to flaccid paralysis) = 1.0; disequilibrium with mild torticollis or tail rigidity = 2.0; continuous torticollis, axial rotation, or abnormal bodily contortions = 3.0. Forelimb weakness was assessed by the animal’s ability to grasp the wire cage top and maintain hold against slight pull. Mild torticollis was scored as a slight tilt of the head to either side. Severe torticollis was marked by involuntary body rotation along the longitudinal axis. Abnormal bodily contortions were defined as asymmetrical bilateral extension of the hindlimbs and/or corkscrew tail.

The cynomolgus macaque received subcutaneous immunizations with an emulsion containing 500 µg rat IgV-MOG in IFA on days 0 and 22 together with additional booster immunizations in IFA containing a mixture of 500 µg rat IgV-MOG and 500 µg macaca IgV-MOG on days 36, 63, and 93 (Table 1). Each immunization or booster was given in a total volume of 0.4 mL as 4 separate 0.1 ml injections in each of the axillary and inguinal areas. The subject was scored at least once daily over the 2-year observation period and three times daily during the phase of active disease. The following clinical scale was used to score EAE in the NHP: loss of finger dexterity, dyscoordination of hand(s)/feet, or repetitive mastication movements, droop in facial features, guarded ambulatory movement, pronounced lack of voluntary movement = 0.5; gross motor dyscoordination during ambulation, apparent sensory disturbance, transient myotonia with uncontrolled balling of hand or foot = 1.0; gross unilateral or bilateral weakness, transient myotonia with full limb involvement including rigid extension paralysis or falling to one side = 2.0; hemi- or paraparesis = 2.5; hemi- or paraplegia = 3.0; quadriplegia and/or somnolence = 4.0; moribund = 5.0.

**Table 1:** The immunization and serum collection schedule for the cynomolgus macaque.

Day		Immunization parameters^a^
0	Serum collection	Primary immunization 500 µg rat IgV-MOG
22	Serum collection	Boost with 500 µg rat IgV-MOG
36	Serum collection	Boost with 500 µg rat IgV-MOG+500 µg macaca IgV-MOG
49	Serum collection	Boost with 500 µg rat IgV-MOG+500 µg macaca IgV-MOG
63	Serum collection	
93	Serum collection	Boost with 500 µg rat IgV-MOG+500 µg macaca IgV-MOG
210	Serum collection	
469	Serum collection	
730	Serum collection	

a The immunization protocol was comprised of a primary immunization in IFA on day 0 followed by 4 booster immunizations in IFA on the designated days. On each day, subcutaneous injections of rat and/or macaca IgV-MOG were given as 4 separate 100 µl injections (total volume of 400 µl) in the left and right groin and axilla. These immunizations did not result in any visible signs of inflammation at the injection sites. Humane euthanasia was performed on Day 730.

### Histological assessment of EAE in rats

After humane euthanasia, the spinal column and brain were removed and were fixed in 10% neutral buffered formalin. Spinal cords were removed from the spinal column after fixation. Standard sections were prepared for each CNS. Standard longitudinal sections were prepared of the entire spinal cord (cervical, thoracic, lumbar, and sacral cord). Standard sections of the brain and brainstem were visualized as an obliquely angled section of the horizontal plane that spanned the prefrontal cortex through the base of the brainstem. Standard sections of the brain and cerebellum were visualized as an obliquely angled section of the horizontal plane that spanned the prefrontal cortex through the posterior lobe of the cerebellum. Sections were stained with hematoxylin and eosin to visualize perivascular mononuclear infiltration or Luxol Fast Blue to visualize demyelination. Regions of demyelination were defined by areas lacking the Luxol Fast Blue myelin stain. CNS lesions were scored by a blinded observer. Sections were imaged with a Leica DFC420C digital camera connected to a Leica DM400B microscope at 25× or 400× total magnification.

### Fluorescent-linked immunosorbent assay analyses

Blood was collected from the tail vein of Lewis rats under isoflurane anesthesia. Similarly, macaca blood was collected from the femoral vein. Serum was harvested after clotting by centrifugation and was stored in aliquots frozen at −80°C. Adherent HEK cells were grown to 60–80% confluence and incubated with a release buffer (2 mM EDTA and 0.5% bovine serum albumin in phosphate buffered saline) for 5–10 minutes. HEK cells were then dispensed into flow tubes at 2×10^5^ cells/tube. HEK that did or did not express full length rat or macaca MOG were incubated with 0.2%–2% titrations (unless designated otherwise) of control serum, immune rat serum, or immune macaca serum. After an initial incubation of 45 minutes, HEK were washed 3 times in Hank’s buffered salt solution supplemented with 1% heat inactivated fetal bovine serum. Rat Ab specific for conformational epitopes of MOG were detected by staining with a secondary allophycocyanin-conjugated goat-anti-rat IgG/M Ab (Southern Biotech, Birmingham, AL) followed by 3 more washes. Binding of macaca anti-MOG immune serum to HEK was detected by use of allophycocyanin-conjugated goat-anti-monkey IgG/M/A, IgG, IgA, or IgM Ab (Brookwood Medical Center, Birmingham, AL). Data were collected by use of a Becton-Dickinson LSRII flow cytometer (Franklin Lakes, NJ) and analyzed by use of FlowJo software (FlowJo LLC, Ashland, OR). An alternative plate-based staining assay was also devised to measure anti-MOG Ab binding. Adherent HEK cells were grown to 60–80% confluence in 96 well microtiter plates. The wells were incubated with designated titrations of anti-MOG sera for 45 minutes, followed by three washes, an incubation with the secondary reagent, another three washes, a brief exposure to the release buffer followed by neutralization of the EDTA with complete RPMI media (containing 10% heat-inactivated fetal bovine serum), and flow analysis on the Becton-Dickenson LSRII flow cytometer with the High Sample Throughput 96 well plate reader. Staining protocols for both tube and plate assays were performed at 4°C.

## Results

### IgV-MOG induces atypical EAE in Lewis rats

**Figure 1:**
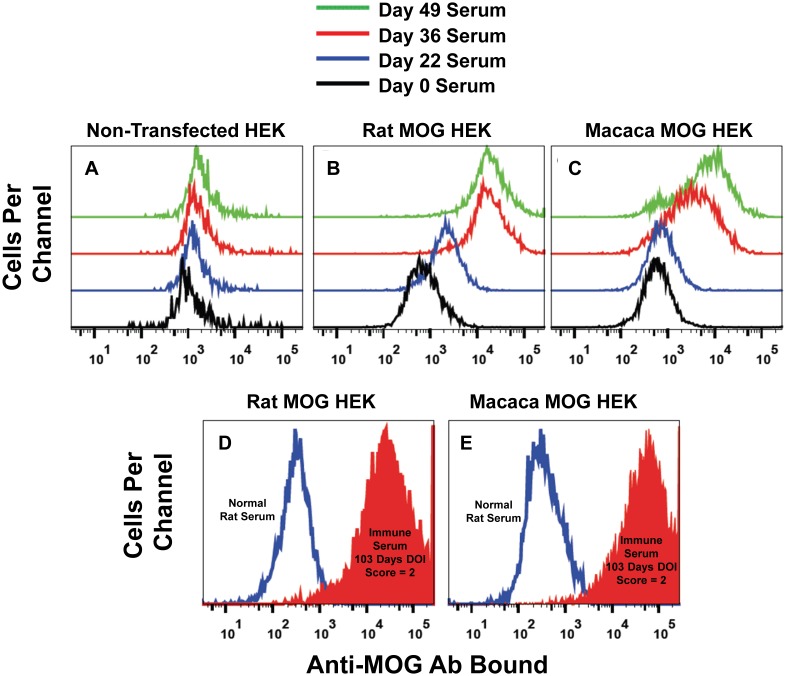
Booster immunizations with IgV-MOG in IFA caused a chronic course of atypical EAE. Seven Lewis rats were immunized with a mixture of 25 µg of GP69-88 and 50 µg of rat IgV-MOG in CFA on day 0. After resolution of a classical monophasic bout of EAE, the same rats were boosted with 200 µg of rat IgV-MOG in IFA on days 45 and 85. Rats were scored daily for clinical signs of EAE. After the IgV-MOG boosts in IFA, the incidence of chronic atypical EAE was 100% (7 of 7 rats). The red arrows (bottom panel) mark the dates of the boosts. Shown are the individual disease courses for the 7 rats. The blue line divides the clinical data into classical (left) and atypical (right) EAE courses.

Previous studies have shown that Lewis rats immunized with GP69-88 in CFA exhibit an acute monophasic course of classical EAE [13], [26]. After spontaneous recovery, rats remain healthy and typically do not exhibit a subsequent relapse of severe EAE. Conversely, immunization of Lewis rats with IgV-MOG induces a mild episode of classical EAE associated with focal demyelination [14]. Pathogenesis appeared to reflect synergy between a weak encephalitogenic MOG-specific T cell response and generation of a MOG-specific, demyelinating Ab. Given that the dominant encephalitogenic region of myelin basic protein elicits strong encephalitogenic T cell responses and that conformational epitopes of MOG elicit demyelinating Ab, preliminary studies were performed to assess the EAE phenotype in Lewis rats co-immunized with both GP69-88 and IgV-MOG (Figure 1). Immunization of seven Lewis rats with 25 µg GP69-88 and 50 µg rat IgV-MOG emulsified in CFA caused a monophasic episode of EAE marked by an ascending flaccid paralysis followed in 3–5 days by a full and spontaneous recovery. This initial bout was essentially identical to what is typically observed in myelin basic protein-induced classical EAE. Following recovery, subsequent boosts of rat IgV-MOG in IFA on days 45 and 85 caused a second bout of chronic ‘atypical’ EAE marked by unusual clinical signs: forelimb weakness without hindlimb involvement, ataxia without flaccid paralysis of the tail, dystonia, rigid asymmetric extension of a hindlimb or forelimb (as opposed to flaccid paralysis), and/or vertigo/disequilibrium and torticollis. The incidence of both classical and atypical disease was 100% (7 of 7 rats), yet the day of onset for atypical EAE was not uniform and the rate of disease progression was varied (Figure 1). Rats were humanely euthanized for weight loss greater than 25%, extreme dehydration for more than 48 hours, or at the conclusion of the experiment on day 125. Despite inconsistencies in the kinetics of disease, these data suggested that a severe course of atypical EAE could be reliably elicited in Lewis rats.

**Figure 2:**
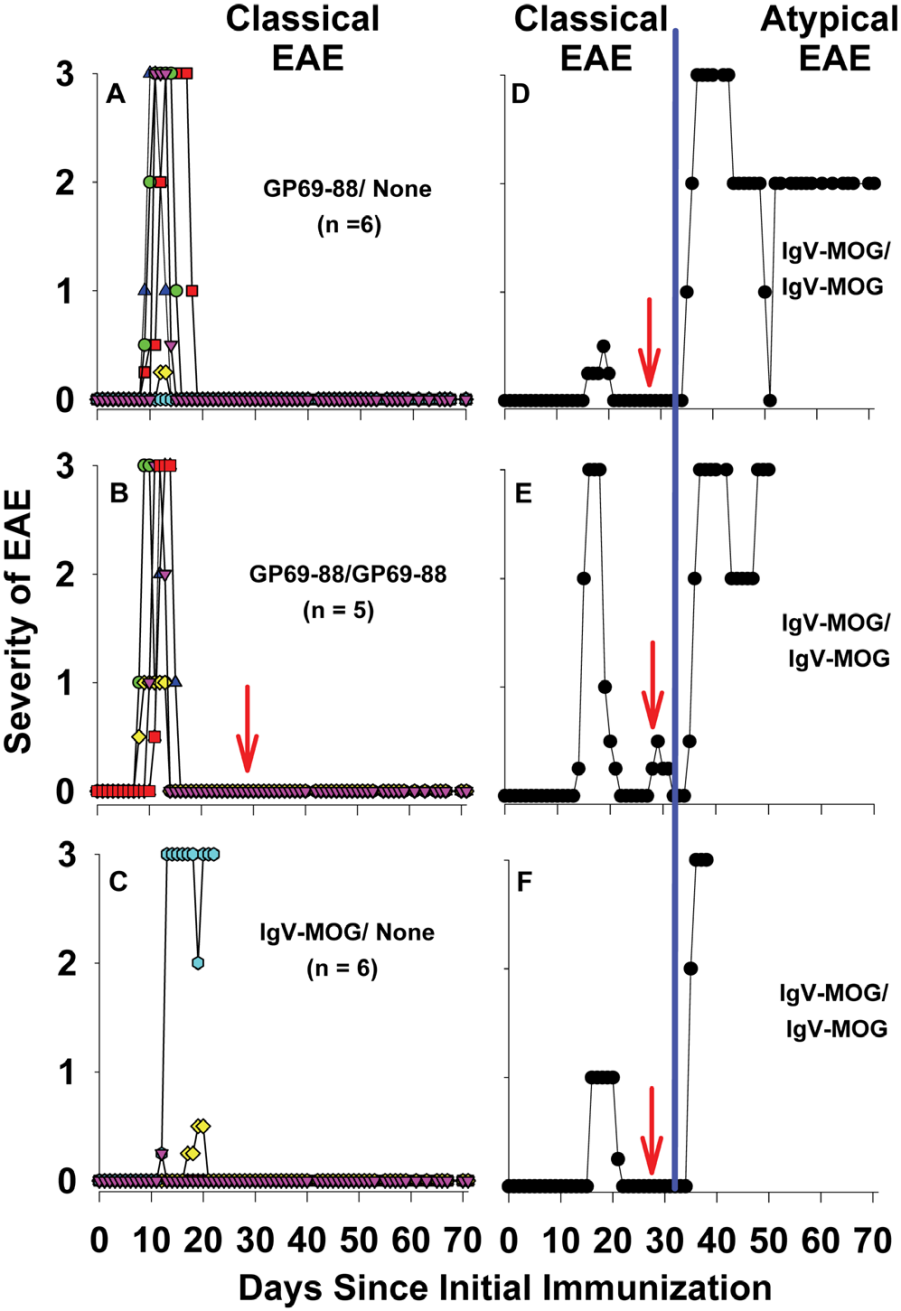
A CFA/IFA prime-boost strategy with IgV-MOG elicited chronic atypical EAE in Lewis rats. Lewis rats were immunized with either 50 µg of GP69-88 or 200 µg of rat IgV-MOG in CFA on day 0. Rats were scored daily for clinical signs of EAE. On day 28, GP69-88-immunized rats were or were not boosted with 50 µg of GP69-88 in IFA. Conversely, rat IgV-MOG-immunized rats were or were not boosted with 200 µg of rat IgV-MOG in IFA. Shown are overlapping traces of disease courses of rats immunized with GP69-88 (A), immunized with GP69-88 and given a subsequent boost (B), or immunized with rat IgV-MOG without a subsequent boost (C). Also shown are atypical disease courses of individual rats immunized and boosted with rat IgV-MOG (D–F; 3 of 4 rats shown). Red arrows indicate the booster immunization (B, D–F). The blue line divides the clinical data into classical (left) and atypical (right) courses (D–F).

To address the minimal immunization requirements to elicit atypical EAE, two groups of rats (n = 12 each) received a primary immunization on day 0 with either 50 µg GP69-88 in CFA or 200 µg IgV-MOG in CFA on day 0 (Figure 2 & Table 2). One GP69-88 immunized rat and one IgV-MOG immunized rat reached a humane endpoint during the first episode of acute classical EAE and thereafter were excluded from the remainder of the experiment. Two IgV-MOG immunized rats developed adjuvant arthritis on day 15 and 19 and were excluded from the experiment. On day 28, rats that were given GP69-88 in CFA were (n = 5) or were not (n = 6) boosted with 50 µg GP69-88 in IFA. Rats that received IgV-MOG in CFA were (n = 4) or were not (n = 5) boosted with 200 µg IgV-MOG in IFA. Rats immunized with GP69-88 in CFA developed classical disease marked by ascending paralysis and spontaneous recovery (Figures 2A & 2B) whereas a subsequent boost of GP69-88 in IFA had no clinical effect (Figure 2B). A single immunization with IgV-MOG in CFA elicited a monophasic episode of classical EAE in 6 of 10 rats but no atypical EAE (Table 2 & Figures 2C–F). One of these rats had severe classical EAE that necessitated humane euthanasia (Figure 2C). After recovery, Lewis rats immunized with IgV-MOG in CFA that were not re-challenged remained healthy for the remainder of the experiment (Figure 2C). In contrast, IgV-MOG/CFA immunized rats boosted with IgV-MOG in IFA exhibited a course of atypical EAE (4 of 4 rats, Table 2). The clinical courses of the 3 most severely afflicted rats are shown in Figure 2D–F. One of these rats exhibited uncontrolled axial rotation (Figure 2F) and was humanely euthanized. A 4^th^ rat (not shown) did not initially develop classical EAE via the first immunization, but after the subsequent booster of rat IgV-MOG in IFA, exhibited monophasic atypical disease from day 42 to 49 characterized by forelimb weakness, ataxia without tail paralysis, and rigid asymmetrical limb extension (maximal score = 1.0). The use of IFA for both IgV-MOG immunization and boosting did not elicit EAE (data not shown). Overall, the main observation was that after spontaneous remission from IgV-MOG/CFA-primed classical EAE, a separate subsequent challenge with IgV-MOG/IFA caused atypical EAE (Figure 2D–F) whereas the parallel strategy did not elicit a relapse with GP69-88 (Figure 2B). These findings provide a protocol to induce atypical EAE in Lewis rats.

**Table 2:** Secondary responses to IgV-MOG elicited chronic atypical EAE in Lewis rats.

Immunization in CFA^a^	Incidence during 1^st^ bout of EAE		Boost in IFA^a^	Incidence during 2^nd^ bout of EAE	
	Classical EAE	Atypical EAE		Classical EAE	Atypical EAE
GP69-88	5 of 6	0 of 6	None	0 of 6	0 of 6
GP69-88	6 of 6	0 of 6	GP69-88	0 of 5	0 of 5
IgV-MOG	3 of 6	0 of 6	None	0 of 5	0 of 5
IgV-MOG	3 of 4	0 of 4	IgV-MOG	0 of 4	4 of 4

a Lewis rats were immunized with 50 µg GP69-88 (n = 12) or 200 µg rat IgV-MOG (n = 12) in CFA on day 0. On day 28, GP69-88-immunized rats were (n = 5) or were not (n = 6) boosted with 50 µg GP69-88 in IFA, and IgV-MOG-immunized rats were (n = 4) or were not (n = 5) boosted with 200 µg rat IgV-MOG in IFA. Rats were scored daily for clinical signs of EAE. Classical and atypical EAE are defined in the Materials and Methods section.

**Figure 3:**
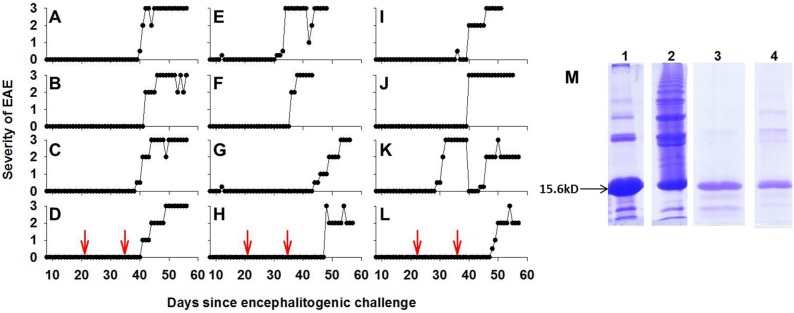
Lewis rats immunized with intra-chain disulfide-linked IgV-MOG developed chronic atypical EAE. Female Lewis rats aged 8 weeks were immunized with 200 µg of rat IgV-MOG (n = 14) in CFA on day 0. These mice were boosted with 200 µg of rat IgV-MOG in IFA on days 21 and 35. Shown are the individual disease courses for the 12 symptomatic rats (A–L) that exhibited atypical EAE. All clinical data were scored based on atypical scoring criteria. The red arrows (bottom panels) mark the dates of the boosts. Panel M shows the IgV-MOG protein preparation before TCEP reduction (Lanes 1–2, reducing vs native SDS-PAGE, respectively) as well as IgV-MOG after TCEP reduction and refolding (Lanes 3–4, reducing vs native SDS-PAGE). These clinical data are described in Table 3. Histological analyses are depicted in Figures 4–6 and Table 4.

**Figure 4:**
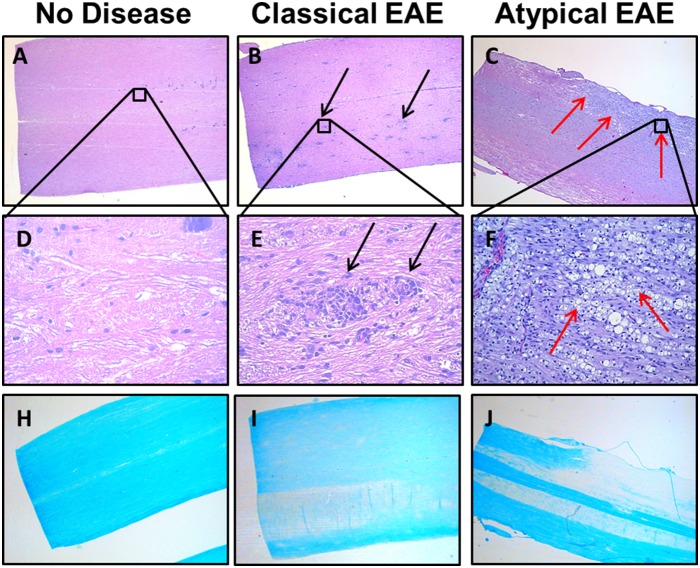
Differential lesion histopathology in the spinal cords of rats afflicted with classical versus atypical EAE. Shown are representative sections of the sacral spinal cord of rats that were healthy (A, D, H) or afflicted with either classical EAE (B, E, I) or atypical EAE (C, F, J). Sections H–J show demyelination via Luxol Fast Blue stain. Red arrows highlight areas of confluent infiltration. Black arrows show focal perivascular infiltration. Sections are from rats shown in Figure 3 and summarized in Tables 3–4.

**Figure 5:**
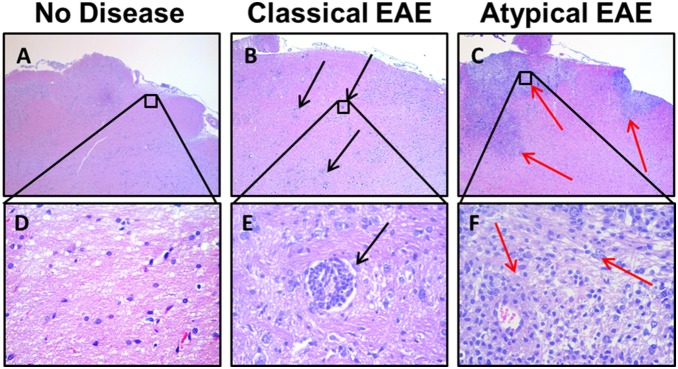
Differential lesion histopathology in the brainstem of rats afflicted with classical versus atypical EAE. Shown are representative sections of the brainstem of rats that were healthy (A, D) or afflicted with either classical EAE (B, E) or atypical EAE (C, F). Red arrows highlight areas of confluent infiltration. Black arrows show focal perivascular infiltration. Sections are from rats shown in Figure 3 and summarized in Tables 3–4.

**Figure 6:**
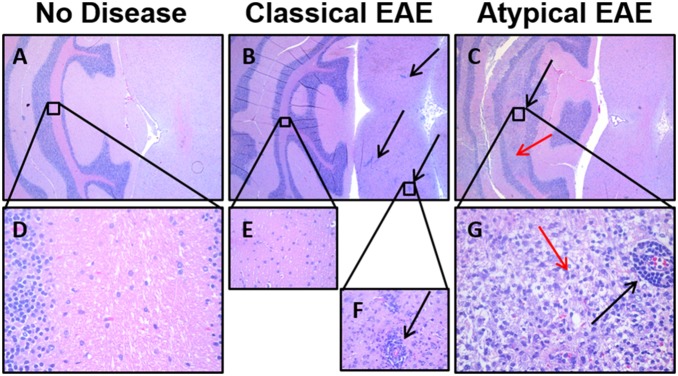
Differential lesion histopathology in the cerebellum of rats afflicted with classical versus atypical EAE. Shown are representative sections of the hindbrain and cerebellum of rats that were healthy (A, D) or afflicted with either classical EAE (B, E–F) or atypical EAE (C, G). Red arrows highlight confluent lesions. Black arrows show focal perivascular infiltration. Sections are from rats shown in Figure 3 and summarized in Tables 3–4.

The experiments shown in Figures 1–2 were repeated based on a refinement of the IgV-MOG preparation method. For the IgV-MOG preparation used in Figure 3, disulfide bridges in IgV-MOG were reduced before the protein was refolded at low concentrations to optimize intramolecular disulfide linkages and to minimize intermolecular disulfide bridges. IgV-MOG contains a single intramolecular disulfide bond. Previously, we noted that during protein extraction and purification, oxidation introduced abnormal crosslinking of IgV-MOG molecules via intermolecular disulfide bonding at the expense of appropriate intra-chain disulfide linkages. Lewis rats (female, n = 20, 8 weeks of age) were immunized with 200 µg of monomeric rat IgV-MOG (n = 14) or 50 µg of GP69-88 (n = 6) in CFA on day 0 (Table 3 and Figure 3). The same groups were respectively boosted with 200 µg of monomeric rat IgV-MOG in IFA or 50 µg GP69-88 in IFA on days 21 and 35. Rats were scored and weighed by a blinded observer for clinical signs of EAE once per day before EAE onset and twice per day after onset. Following a second boost of IgV-MOG/IFA on day 35, the incidence of chronic atypical EAE was 92.3% (Table 3). One rat from this group had severe adjuvant arthritis and was euthanized on day 22 prior to EAE development. An additional rat did not exhibit signs of EAE throughout the experiment. Figure 3 depicts the clinical courses of atypical EAE for the 12 symptomatic rats (Figure 3, A–L). The mean and median day of onset was day 39.3 and 40, respectively. Figure 3M (lane 4) shows a 15% SDS-PAGE gel of the protein preparation used for immunization. Protein preparations before (lanes 1–2) and after (lanes 3–4) TCEP reduction are shown under reducing (lanes 1, 3) and non-reducing (lanes 2, 4) conditions. TCEP-HCl was used to reduce all disulfide bonds before refolding of the protein at low protein concentrations to optimize intra-chain disulfide linkages. The use of TCEP-HCl to optimize intrachain disulfide linkage in rat IgV-MOG is relevant to Tables 3–4 and Figures 3, 4, 5, 6. Lane 4 of Figure 3M represents the folded, native protein used for immunization, and Table 3 lists the incidence of classical and atypical EAE for each group. Taken together (Figures 1–3), these data indicate that rat IgV-MOG can be used to reliably induce atypical EAE in Lewis rats by use of a CFA immunization/IFA booster strategy.

**Table 3:** An intrachain disulfide-linked IgV-MOG protein elicited chronic atypical EAE in rats.

Immunization in CFA^a^	Incidence of EAE after 1^st^ immunization		Incidence of recovery	Boosts in IFA	Incidence of EAE after boosts		Incidence of recovery
	Classical EAE	Atypical EAE			Classical EAE	Atypical EAE	
GP69-88	6 of 6	0 of 6	6 of 6	GP69-88	0 of 6	0 of 6	N/A
IgV-MOG	2 of 13	0 of 13	2 of 2	IgV-MOG	0 of 13	12 of 13	0 of 12

a Lewis rats were immunized with 50 µg GP69-88 (n = 6) or 200 µg rat IgV-MOG (n = 14) in CFA on day 0. On days 21 and 35, rats were boosted with the same respective antigen (50 µg GP69-88 or 200 µg rat IgV-MOG) in IFA. Immunization and boosting with IgV-MOG were performed with an intrachain disulfide-linked IgV-MOG protein. Rats were scored daily for clinical signs of EAE. Figure 3 and Table 3 portray the same experiment. Recovery of rodents was defined as remission to a clinical score of 0 after an episode of paralytic disease.

**Table 4:** Summary of CNS histological analyses.

Sensitizingantigen^a^	Incidence ofclassical EAE	Incidence ofatypical EAE	Mean clinicalscore	Predominantlesion type^b^
GP69-88	4 of 4	0 of 4	3.0	Focal
IgV-MOG	0 of 10	10 of 10	3.0	Confluent

a Histological assessment of classical EAE was assessed in 4 Lewis rats immunized with 50 µg GP69-88 in CFA. Histological assessment of atypical EAE was based on 10 Lewis rats immunized with 200 µg rat IgV-MOG in CFA and boosted with MOG-IgV/IFA on days 21 and 35. Rats were euthanized at peak disease.

b Representative sections are shown in Figures 4–6. In rats with classical EAE, approximately 100% of the lesion area in the CNS consisted of punctate areas of mononuclear perivascular infiltration. In rats with atypical EAE, nearly 100% of the lesion area in the CNS was comprised of confluent lesions which were readily discernable by the unaided eye on H&E sections as a dark blue in contrast to the light purple of surrounding normal tissue. Confluent lesions typically involved areas greater than 1 mm^2^ and ranged from 1 mm to greater than 1 cm in one dimension, particularly in the spinal cord or at the base of the brainstem.

### Large confluent demyelinating lesions are predominant in atypical EAE

Histological analyses of these rats (Figure 3) showed that classical and atypical forms of EAE were associated with qualitatively different types of inflammatory CNS lesions (Table 4). Classical EAE was associated with numerous focal inflammatory lesions. In contrast, atypical EAE was associated with large confluent lesions marked by extensive demyelination and by vacuolation (Figures 4, 5, 6). In both classical and atypical disease, the majority of lesions were in the spinal cord (Figure 4) and in the base of the brain stem (Figure 5). One rat with atypical EAE also had an extensive cerebellar lesion (Figure 6). Healthy rats were devoid of CNS lesions (Figures 4, 5, 6A, 4–6D) and had normal myelination (Figure 4H). Figure 4 portrays representative spinal cord sections. Classical EAE was associated with numerous focal lesions (Figure 4B & 4E) and diffuse areas of demyelination coincident with perivascular infiltration (compare Figures 4B & 4I). Atypical EAE was marked by confluent lesions encompassing large areas of infiltration, vacuolation, and necrotic damage (Figures 4C & 4F). These confluent lesions were marked by a nearly complete absence of myelin (compare Figures 4C & 4J) and the presence of large vacuoles. Confluent lesions often spanned several longitudinal centimeters of spinal cord and were bilateral or unilateral in regard to the width of the cord. Confluent lesions were also noted for pathological changes in gray matter neurons (not shown). The cauda equina appeared normal. Figure 5 portrays representative lesions in the brainstem. Again, classical EAE was associated with focal inflammatory lesions (Figure 5B & 5E) whereas the brainstems of rats afflicted with atypical EAE all had extensive confluent lesions (Figure 5C & 5F). Figure 6 shows sections of the posterior brain and cerebellum. In classical EAE, sparse focal lesions were seen in caudal brain (Figure 6B & 6F) whereas the cerebellum lacked inflammatory lesions (Figures 6B & 6E). In one rat with atypical EAE, a large confluent lesion was noted in the cerebellum (Figures 6C & 6G). In either classical or atypical EAE, no lesions were noted in rostral brain regions. Initial histological analysis of rats used in Figures 1–2 revealed evidence for a greater lesion load in the rostral brain in atypical EAE compared to classical EAE. However, such a difference in lesion distribution was not evident for the analysis of rats shown in Figure 3. Overall, these findings revealed a qualitative difference in lesion type within the CNS of rats afflicted with classical versus atypical EAE.

### IgV-MOG induces atypical EAE in *Macaca fascicularis*

**Figure 7:**
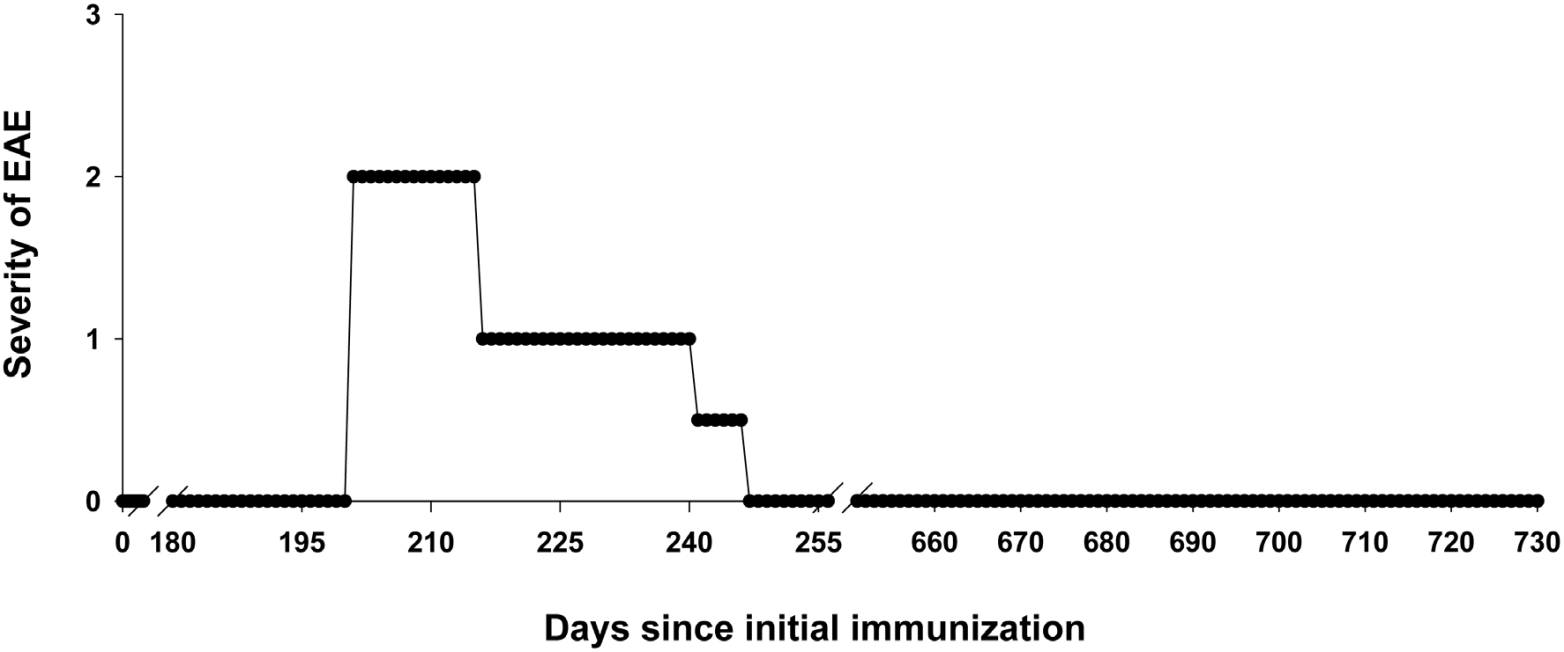
Repeated booster immunizations of IgV-MOG in IFA caused an unusual form of EAE in a cynomolgus macaque. A cynomolgus macaque was immunized with 500 µg rat IgV-MOG in IFA on days 0 and 22. Booster immunizations that consisted of a mixture of 500 µg rat IgV-MOG and 500 µg macaca IgV-MOG in IFA were given on days 36, 63, and 93. Data represent the full time course of the experiment. After recovery on day 248, the subject did not exhibit additional signs of EAE. Table 1 provides a timeline for the experimental approach.

Given that IgV-MOG was pivotal for the induction of atypical EAE in Lewis rats, a relevant question was whether a similar strategy based on repeated boosting with IgV-MOG in IFA would also elicit atypical EAE in NHP. We had one male cynomolgus macaque available for this project. The cynomolgus macaque was immunized with 500 µg of rat IgV-MOG and then four boosts of either rat IgV-MOG alone or a combination of rat and macaca IgV-MOG (Table 1). All antigen injections were in IFA. The subject was directly observed once per day until disease onset and then 3 times per day in ∼30 minute sessions during overt disease. The NHP exhibited a single episode of EAE with an onset on day 201 after the initial immunization and 108 days after the final boost (Figure 7, Table 1).

On day 201, the onset of EAE was abrupt and was marked by gross motor dyscoordination in both legs, including clumsy ambulation and awkward movement about the enclosure, falling to one side, use of arms to support walking and sitting, slow deliberate and guarded movements, apparent sensory disturbances, lack of bilateral hand strength and finger dexterity, difficulty climbing to the perch or reaching food at the top of the enclosure, involuntary but transient balling of hand and foot, and repetitive chomping motion in jaws. These clinical signs persisted with slight improvement through day 207.

On days 208–215, the subject continued to show gross motor dyscoordination, including a wobbly gait, use of the arms to brace against the enclosure during ambulation, slow hesitant movements, difficulty in climbing onto the perch or reaching the top of the enclosure, occasional but substantial weakness on the right side including the right leg, and continued difficulty with finger dexterity. One of the distinct clinical signs that became apparent during this period was a marked transient myotonia associated with any sudden arousal, which was particularly evident at the beginning of each observation period. These episodes of severe disability transpired over 1–2 minutes and then were absent during the remainder of the observation period. These episodes of myotonia had variable presentation, including falling to one side, gross motor dyscoordination during ambulation, rigid extension paralysis of one leg or arm, and/or involuntary balling of a foot or hand. Overall, the myotonic episodes resembled a neuromuscular intention spasm of a brief duration followed by completely normal function.

On days 216–221, the subject showed gradual improvement. The main signs included slow deliberate but coordinated motion punctuated by mild bouts of dyscoordination during ambulation. Finger coordination was improved but not yet normal and was associated with improved hand strength. The myotonic episodes represented the most evident sign of disability. On days 222–242, the major neurological disability included continued episodes of arousal-induced frozen myotonia which gradually became less frequent, less severe, and of shorter duration. On days 243–247, the subject exhibited mild, transient dyscoordination of hands and feet but was otherwise normal. On day 248, the subject was scored as normal (score of 0) and did not exhibit any additional signs of disability to the end of the observation period at day 730.

Through the entire course of observation, the subject did not exhibit flaccid paralysis or overt clinical signs consistent with classical EAE such as progressive paralysis or an ascending flaccid paralysis of the tail or hindlimbs. Nor did the subject show any indication of hyperacute encephalitis as has been noted in other NHP models of EAE [18]–[21], [27].

### Atypical EAE is associated with IgG specific for extracellular, conformational epitopes of MOG

**Figure 8:**
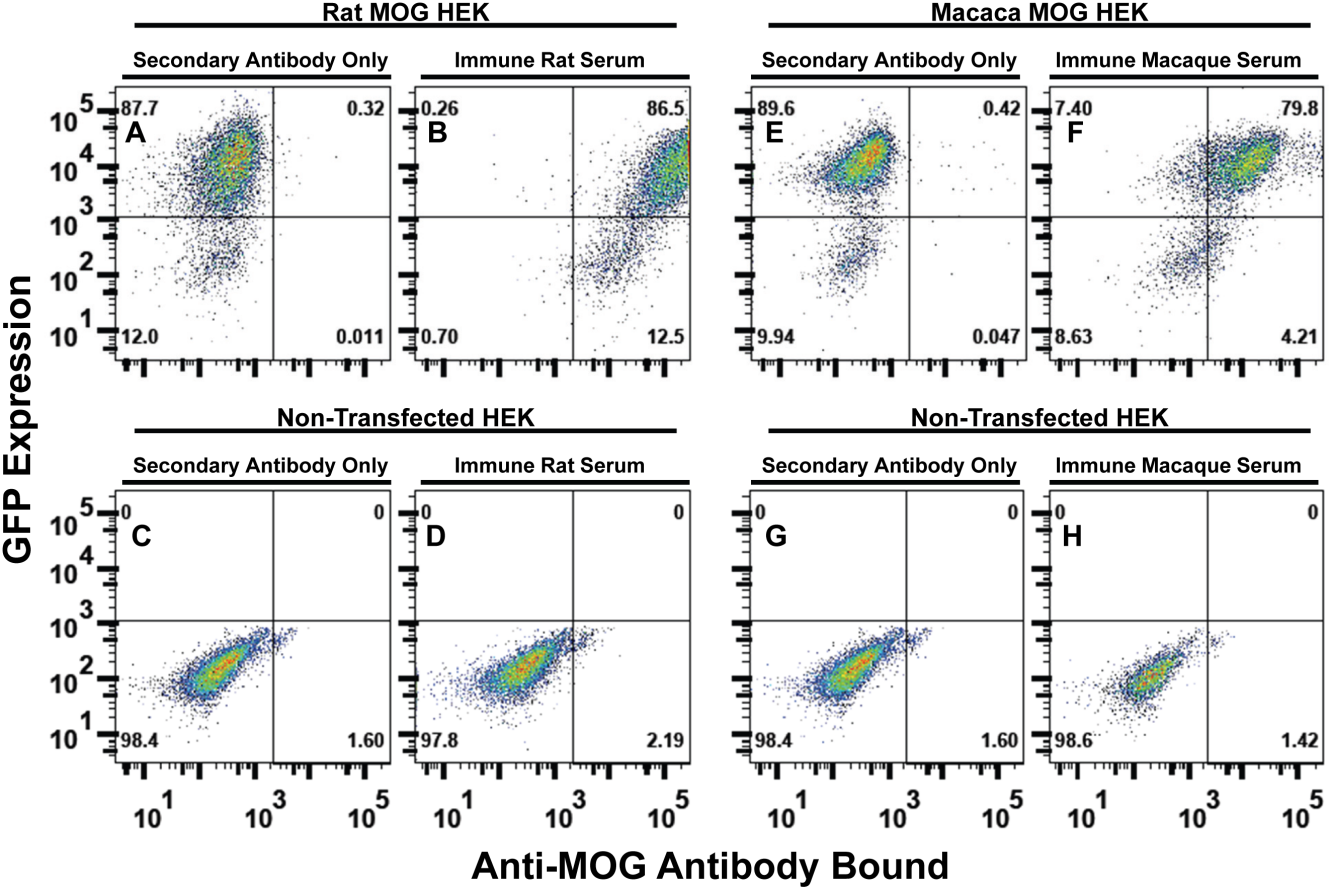
Flow cytometric assay of serum Ab specific for conformational epitopes of IgV-MOG. Rat MOG HEK (A–B), macaca MOG HEK (E–F), or naive HEK (C–D, G–H) were incubated with (B, D, F, H) or without (A, C, E, G) anti-MOG immune serum, including rat anti-rat MOG immune serum (B, D) or macaca immune antisera (F, H). The rat serum was from a rat primed with 200 µg IgV-MOG/CFA, boosted on day 25 with 200 µg IgV-MOG/IFA, and drawn on day 36 when the rat was exhibiting clinical signs of atypical EAE including rigid hindlimb extension and forelimb weakness. The macaca serum was obtained on day 36 as described in Table 1. After labeling with primary Ab, rat MOG HEK were washed and incubated with an allophycocyanin-conjugated goat-anti-rat IgG/M secondary reagent, and macaca MOG HEK were washed and stained with an allophycocyanin-conjugated goat-anti-monkey IgG/M/A Ab. The *y*-axis shows expression of GFP, and the *x*-axis represents the amount of anti-MOG Ab bound to the HEK cell surface. Analyses were of individual serum samples, not pooled sera.

**Figure 9:**
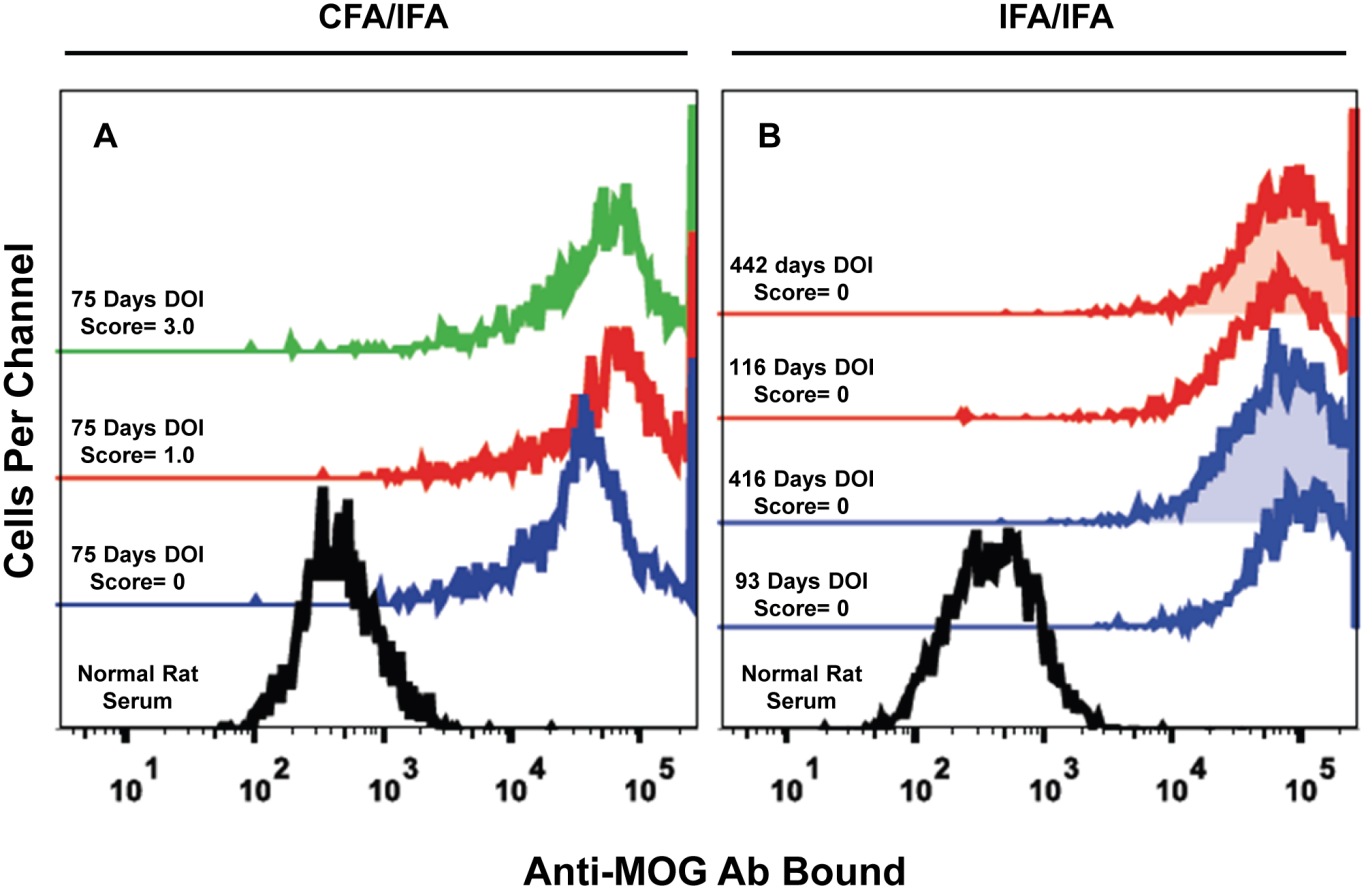
Lewis rats immunized with IgV-MOG developed Ab specific for conformational epitopes of MOG. (A) Lewis rats were immunized with IgV-MOG in CFA, boosted with IgV-MOG in IFA, and sera were taken on day 75. Clinical courses of donor rats are shown in Figure 1 (numbered 1–7 top to bottom). Shown are GFP^+^-gated rat MOG HEK cells stained with: Black trace = normal rat serum; Blue trace = rat #2 with clinical score of 0; Red trace = rat #1 with clinical score of 1.0; Green trace = rat #3 with score of 3.0. (B) Lewis rats were immunized with IgV-MOG in IFA via the same antigens and schedule as designated for the NHP in Table 1. These rats (n = 3) did not exhibit clinically evident EAE. Shown are histograms of GFP^+^-gated rat MOG HEK cells labeled with normal rat serum (black trace) or immune serum from two separate asymptomatic rats (red versus blue) taken 442, 116, 416, and 93 days (top to bottom) after the day of immunization (DOI). Shaded traces indicate at least one year since DOI. Labeled cells were detected with an allophycocyanin-conjugated secondary goat-anti-rat IgG/M secondary Ab. Analyses were of individual serum samples, not pooled sera.

**Figure 10:**
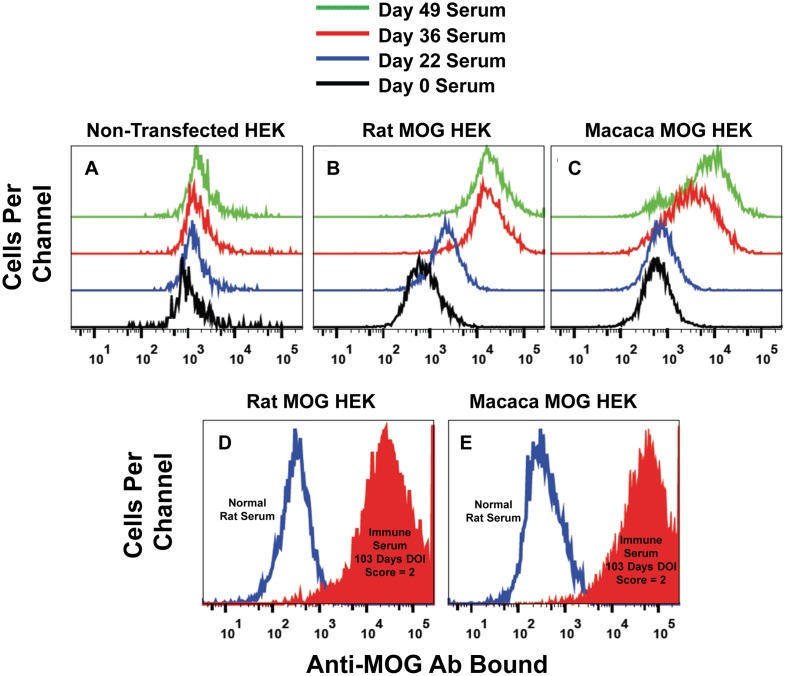
Immunization of a cynomolgus macaque with IgV-MOG elicited Ab specific for conformational epitopes of MOG. Non-transfected HEK (A), rat MOG HEK (B), or macaca MOG HEK (C) were stained with pre-immune serum (day 0 serum) or with macaca serum collected on days 22, 36, or 49 as described in Table 1. Shown are GFP^+^ cells stained with the designated serum and an allophycocyanin-conjugated goat-anti-monkey IgG/M/A Ab. Also shown are histograms of GFP^+^-gated rat MOG HEK (D) or macaca MOG HEK (E) cells stained with normal rat serum (blue trace) or immune rat serum (filled red trace). The immune serum was from an IgV-MOG immunized Lewis rat exhibiting atypical EAE (i.e., rat #6 of Figure 1 taken on day 103 with a score = 2.0). Ab binding was detected using an allophycocyanin-conjugated goat anti-rat IgG/M Ab.

**Figure 11:**
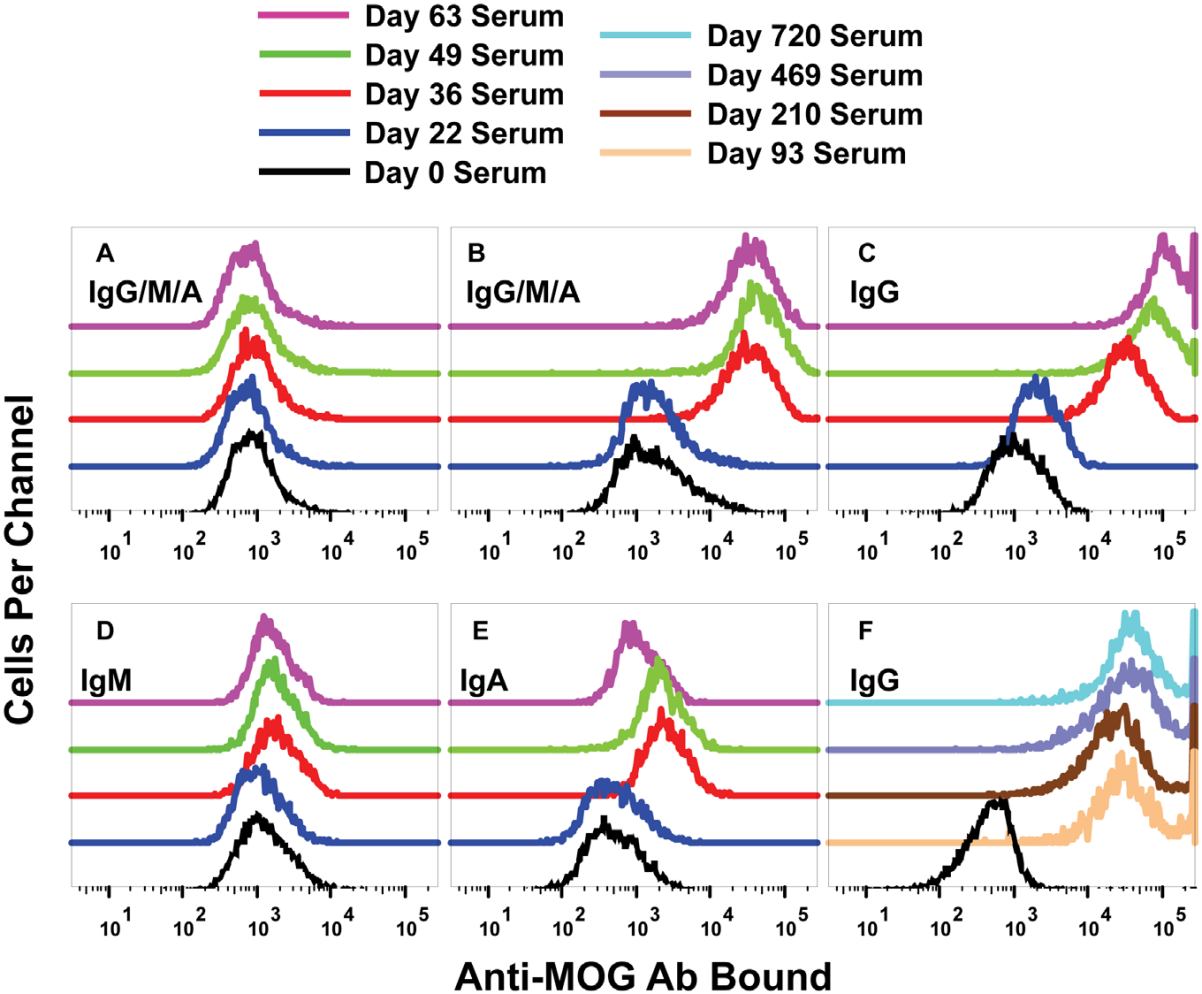
The isotype of the macaca anti-MOG Ab was IgG. Shown are GFP^−^ non-transfected HEK (A) or GFP^+^ macaca MOG HEK (B–F) that were stained with macaca serum collected on days 0, 22, 36, 49, or 63 (A–E) and 93, 210, 469, or 730 (F) post-immunization as described in Table 1. HEK were subsequently stained with an allophycocyanin-conjugated secondary Ab specific for macaca Ab isotypes, including anti-IgG/M/A (A–B), anti-IgG (C, F), anti-IgM (D), anti-IgA (E).

**Figure 12:**
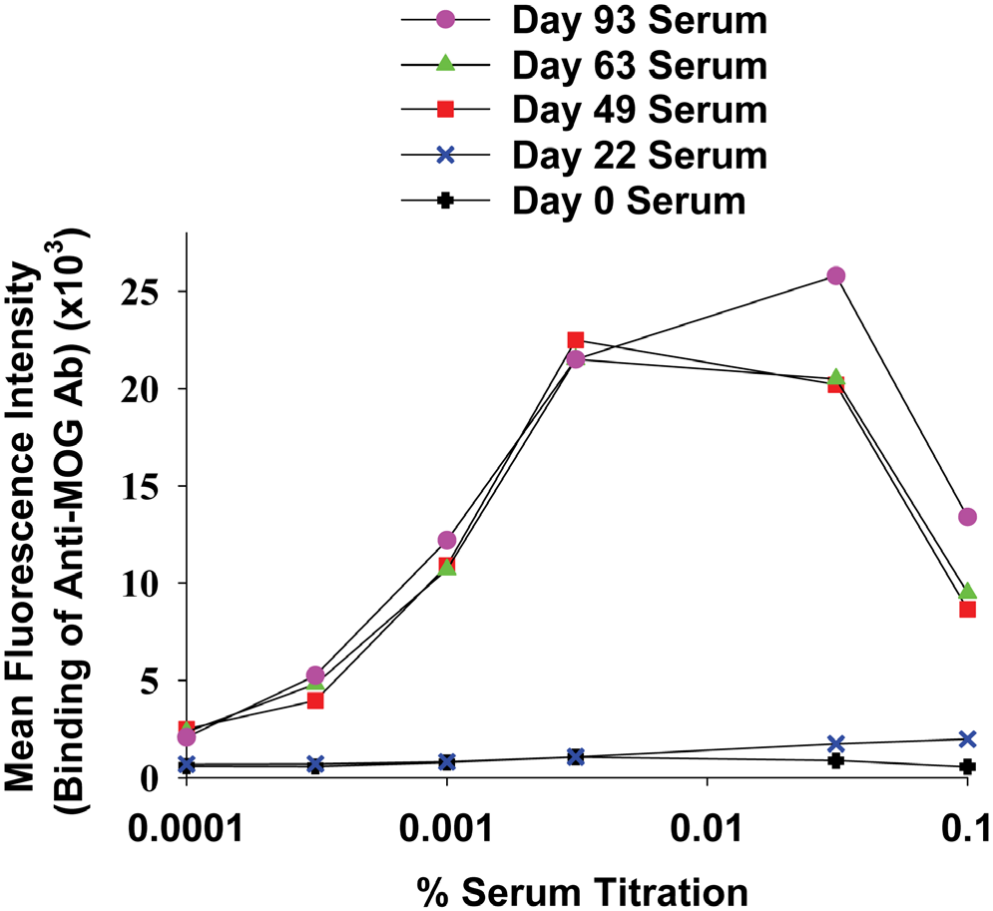
Anti-MOG IgG in macaca immune serum was a high-titer Ab. Macaca MOG HEK were stained with designated titrations of macaca serum (x-axis; 0.1 = 0.1% or 1/1000; 0.0001 = 1/10^6^) collected on days 0, 22, 49, 63, or 93 (Table 1). A subsequent stain with an allophycocyanin-conjugated anti-macaca IgG secondary reagent was used to detect surface bound anti-MOG Ab.

Atypical EAE in Lewis rats and the cynomolgus macaque was associated with high titer Ab specific for extracellular, conformational epitopes of MOG (Figures 8, 9, 10, 11, 12). Fluorescence-linked immunosorbent assays were used to measure serum Ab of IgV-MOG immunized animals. Briefly, HEK were derived that expressed full length rat MOG (rat MOG HEK) or full length macaca MOG (macaca MOG HEK) with GFP. Rat and macaca MOG HEK expressed GFP (Figures 8A & 8E, respectively) and non-transfected HEK lacked GFP expression (Figure 8C & 8G, respectively). Stably transfected HEK bound anti-MOG Ab in immune serum, which was detected by fluorochrome-conjugated secondary Ab against rat or macaque immunoglobulin. As shown in Figure 8A & B, when incubated with an immune serum from IgV-MOG sensitized Lewis rats, rat MOG HEK exhibited an approximate 350-fold enhancement in relative mean fluorescent intensity (MFI) compared to the same cells incubated with a non-immune serum. Non-transfected HEK incubated with the immune serum did not bind anti-MOG Ab (Figures 8C & 8D). Similarly, macaca MOG HEK incubated with immune serum from the IgV-MOG sensitized macaque exhibited 75-fold greater MFI compared to the same cells treated with a non-immune serum (Figures 8E & 8F, respectively). Non-transfected cells did not bind the anti-MOG Ab from immune serum (Figures 8G & 8H, respectively). The rat immunization protocol was associated with higher Ab titers (Figure 8B) compared to what was achieved with the NHP protocol (Figure 8F). Overall, these studies showed that immunization with IgV-MOG derived from a prokaryotic expression system elicited high titer Ab that cross-reacted with full length MOG on HEK. Thus, Ab against soluble non-glycosylated IgV-MOG cross-reacted with native glycosylated cell-surface MOG proteins, presumably via shared conformational epitopes.

### Humoral immunity to IgV-MOG in Lewis rats

Subsequent analyses showed that Lewis rats immunized and boosted with IgV-MOG had high titers of anti-MOG Ab compared to non-immunized naive rats (Figure 9). Immune serum obtained from asymptomatic (Figure 9A, blue trace) and afflicted (Figure 9A, red & green traces) rats 75 days after initial sensitization showed similarly high levels of anti-MOG Ab. Lewis rats immunized and boosted with IgV-MOG in IFA via an IFA-IFA prime-boost strategy did not develop EAE, yet these rats also produced anti-MOG Ab at titers similar to those in diseased rats (Figure 9B). Together, these findings provide evidence that anti-MOG Ab preceded disease development but were not sufficient to induce EAE. Serum collected from the same IgV-MOG/IFA-immunized rats more than one year after the initial sensitization showed essentially the same level of Ab binding (Figure 9B, shaded traces) despite the absence of additional booster immunizations. These data indicate the serum levels of anti-MOG Ab were maintained over substantial periods of time.

### Humoral immunity to IgV-MOG in a NHP

Immunization of the cynomolgus macaque resulted in a high titer of anti-MOG Ab production as assessed by the fluorescence-linked immunosorbent assay. Non-transfected HEK lacked reactivity to all serum preparations tested (Figure 10A). Both rat and macaca MOG HEK bound Ab in macaca immune serum collected on days 36 and 49 but did not bind Ab in pre-immunization serum collected on day 0 (Figures 10B & 10C). Anti-rat MOG and anti-macaca MOG Ab titers increased following a booster immunization with rat IgV-MOG on day 22 and the “rat IgV-MOG + macaca IgV MOG” immunization on day 36 (Figures 10B & 10C). Macaca immune serum reacted strongly with rat MOG (Figure 10B), and rat immune serum reacted extensively with both rat MOG (Figure 10D) and macaca MOG (Figure 10E). These data indicate that both rat and macaca immune sera exhibited extensive cross-reactivity between the rat and macaca MOG proteins. This extensive cross-reactivity was consistent with the close homology of the two MOG species that were distinguished by only 14 amino acid substitutions in the 123 amino acid span of the IgV domain.

To assess the isotype produced by the cynomolgus macaque, non-transfected HEK (Figure 11A) or macaca MOG HEK (Figures 11B–F) were incubated with macaque immune serum in a primary incubation followed by a secondary incubation with goat-anti-monkey IgG/M/A (Figures 11A & 11B), IgG (Figure 11C), IgM (Figure 11D), or IgA (Figure 11E). The results showed that the cynomolgus macaque predominantly produced IgG Ab specific for MOG (Figure 11C). Non-transfected cells did not bind Ab (Figure 11A). The anti-MOG IgG signal was 300-fold greater than the MFI values of controls lacking primary Ab. Anti-monkey IgM and anti-monkey IgA revealed binding levels that were approximately 5-fold greater than negative control samples, but these binding activities were apparent only in the day 36 and day 49 sera and were absent in the day 63 serum (Figures 11D & 11E). Thus, IgM and IgA anti-MOG Ab appeared transiently during the first two months of immunization before the isotype of the humoral response completely switched to IgG (compare Figure 11C & Figures 11D–E). No additional boosters were given after day 93, yet anti-MOG IgG Ab levels remained essentially constant in the serum until day 730 at the end of the observational period (Figure 11F).

The fluorescence-linked immunosorbent assay was not only useful for the qualitative detection of anti-MOG IgG, the assay was also useful for the quantitative measurement of relative anti-MOG IgG titers (Figure 12). In the experiment represented by Figure 12, a plate-based assay was used instead of a tube-based assay (Figures 8, 9, 10, 11). A plate-based staining assay had two main advantages; increased sensitivity for detection of anti-MOG Ab and increased throughput for analysis of multiple samples. The increased sensitivity for Ab-binding activity most likely reflected faster processing time and more favorable dissociation/association kinetics during washing due to decreased wash volumes. Each wash for the tube assay represented an approximate 1/30 dilution whereas each wash for the plate assay represented an approximate ¼ dilution. The plate assay enabled analysis of 96 samples in approximately 20–30 minutes. Three distinct zones were noted in assays that measured dilution of immune serum from a titration of 10^−3^ to a titration of 10^−6^ (i.e., 0.1 to 0.0001 on the *x*-axis) including an antigen-excess zone, a saturable zone of equivalence, and a prozone. From this bell-shaped curve, the antigen excess zone provided an accurate measure of the amount of high affinity IgG in the immune serum. These data revealed that half-maximal binding was detected at a titration of approximately 10^−5^ which provides evidence of high titers of anti-MOG IgG. Saturable binding was observed at titrations of 10^−3.5^ to 10^−4.5^ which most likely reflected bivalent interactions of anti-MOG IgG with cell surface MOG. A distinct prozone was noted at a titration of 10^−2^. The relative lack of signal at this titration may reflect competition of anti-MOG IgG for a limited amount of cell surface MOG, resulting in a preponderance of monovalent IgG interactions with cell surface MOG. In this case, Ab bound via monovalent interactions would predictably lack the avidity to remain bound during the subsequent washes and may be lost by dissociation. Alternatively, the prozone effect may be due to carryover of soluble (non-bound) anti-MOG Ab from the first incubation. Overall, these data showed that serum collected on day 0 and day 22 lacked anti-MOG IgG. After the first boost on day 22, the NHP showed sero-conversion and had uniformly high titers of anti-MOG IgG in all sera collected thereafter.

## Discussion

This study has three lines of significance. First, we established a novel and clinically significant model of atypical EAE in Lewis rats (Figures 1, 2, 3). The model was robust in that the protocol elicited severe paralytic signs. The model was reliable in that the incidence of atypical EAE was usually 100% after one or two boosts with IgV-MOG in IFA. The model was not complicated by a genetic deficiency of the IFN-γ pathway. This model should enable development of novel therapeutics in a system that is mechanistically unique from classical EAE. An optimal model would be comprised of a primary immunization with 200 µg rat IgV-MOG in CFA to elicit monophasic EAE, and after full recovery from classical EAE, secondary booster immunizations with 200 µg IgV-MOG in IFA on approximately days 21 and 35. Although we did not systematically study the timing of the secondary immunizations, our view is that booster immunizations in this timeframe would be optimal for a perspective of cost, expediency, and practicality. The ability to test therapeutics in both atypical and classical models of EAE may reveal therapeutics that could have broad efficacy in mechanistically diverse forms of MS. Because clinical expression of MS relapse seldom resembles classical EAE, the atypical model may have special relevance for identifying therapeutics that can modulate diverse forms of MS.

Second, the study provided evidence that this approach (immunization of IgV-MOG in IFA) also has the potential to elicit a complex neurological syndrome in a cynomolgus macaque (Figure 7). The observation that the subject did not exhibit an acute fatal disease or an ascending flaccid paralysis provided suggestive evidence that this immunization protocol with macaca/rat IgV-MOG in IFA may represent a suitable approach for induction of a disease that more closely mimics MS than other NHP models of EAE.

Third, the study revealed relationships among (a) immunization with a non-inflammatory adjuvant (IFA rather than CFA) (Figures 1, 2, 3, 7), (b) humoral immunity against conformationally intact determinants of IgV-MOG (Figures 8, 9, 10, 11, 12), (c) continuous confluent plaques of severe demyelination in spinal cord and brainstem (Figures 4, 5, 6), and (d) a complex atypical neurologic disease that more closely mimics the clinical heterogeneity of MS than the stereotypic ascending paralysis of classical EAE. The association of the immunization strategy with anti-MOG humoral immunity and atypical EAE were important in both rat and macaca models of EAE. Indeed, the IFA boost strategy of repeated IgV-MOG immunization resulted in high titers of anti-MOG IgG, as tested by the fluorescent-linked immunosorbent assay analyses in 100% of animals subjected to this immunization protocol. Production of anti-MOG Ab preceded onset of EAE and was invariably correlated with the presence of EAE in all animals of this study. The presence of anti-MOG IgG however was not sufficient for EAE because the IFA-IFA prime boost strategy elicited high titer Ab but without EAE (Figure 9).

### Conformational determinants of IgV-MOG may have a special role in atypical EAE

Induction of humoral immunity against globular IgV-MOG and use of a non-inflammatory adjuvant (IFA) represents a rational experimental approach for initiation of atypical EAE. Globular IgV-MOG is considered a substantially more potent B cell immunogen than linear peptides of MOG because multivalent B cell epitopes in the globular protein are all physically linked to the intrinsic carrier T cell epitopes within the protein. Linear peptides however may or may not contain a physically linked carrier peptide. Thus, IgV-MOG would predictably drive a greater diversity and frequency of B cell clones and thereby stimulate a more potent humoral immune response against MOG. The increased frequency of IgV-MOG specific B cells would culminate in higher frequencies of MOG-specific B cell antigen presenting cells (APC) and highly efficient B cell-mediated presentation of MOG. In contrast to myeloid macrophage and dendritic cell APC, B cells drive polarized Th2 cells and down-regulate IFN-γ dominated Th1 immunity particularly when the immunogen is administered in IFA rather than CFA. As discussed below, the lack of IFN-γ is a critical variable favoring atypical EAE.

Conformational determinants of IgV-MOG also have a special role in the effector phase of EAE. Several studies showed that autoreactive T cell-mediated CNS responses to MOG, myelin basic protein, or other CNS myelin proteins act synergistically with demyelinating Ab specific for conformational determinants of MOG to cause extensive plaques of demyelination and aggravated neurologic disease in rats [28]–[33]. T cell-mediated, myelin-specific inflammation may not be sufficient to overtly cause neurologic disease due to many factors such as inefficient T cell antigen recognition or low CNS concentrations of the target antigen. Thus, T cell-mediated responses may be sufficient for perivascular infiltration of mononuclear cells but may lack the potency to drive the continued invasion of the CNS parenchyma that is necessary for severe paralysis. Nonetheless, subclinical perivascular infiltration of CNS-reactive T cells appears sufficient to cause permeation of the blood brain barrier to enable diffusion of Ab and complement into the CNS parenchyma. The binding of IgG to native MOG on the surface of compact myelin is believed to fix complement that in turn triggers the release of anaphylatoxins followed by chemotactic migration and activation of polymorphonuclear and mononuclear phagocytes that engulf opsonized myelin in the CNS [34]–[37]. The consequence is phagocytosis of opsonized myelin lamellae and axonal conduction deficits. Ab-mediated demyelination is dependent upon recognition of conformational determinants on the extracellular domain of MOG, as opposed to recognition of linear continuous determinants of MOG. That is, immunization with conformational MOG epitopes is needed to elicit antibodies that are cross-reactive with native transmembrane MOG in the CNS [38]. For this reason, immunization with synthetic peptides of MOG is not pathogenic and appears epiphenomenal to Ab-dependent models of EAE because Ab against linear epitopes of MOG lack crossreactivity with transmembrane MOG in the CNS.

### Mouse and rat models of atypical EAE

Mouse models of ‘rotatory’ EAE are characterized by idiosyncratic clinical signs qualitatively distinct from classical caudo-rostral progression of flaccid paralysis [7]–[11]. Atypical disease is also marked by an irregular disease progression, inter-mouse heterogeneity in presentation of clinical signs, and in some cases, a significantly delayed onset (∼6 weeks) [11]. Interestingly, a given encephalitogenic peptide induced either classical or atypical EAE phenotypes in different mice of the same syngeneic cohort. Currently, mouse models of atypical EAE lack the predictability of the Lewis rat model and are therefore less suitable for the study of therapeutics for MS. In contrast to these models, the rat model described in Figures 1, 2, 3 and Tables 2–3 resulted in an atypical disease with nearly 100% incidence.

The most reliable models of atypical EAE have been described in IFN-γ deficient mice. Balb/c mice deficient in the IFN-γ signaling pathway are more susceptible to atypical EAE induction, yet mice within the same cohort exhibited different forms of disease [8]. IFN-γ receptor knockout mice show a similar phenotype [39], [40]. Atypical EAE, particularly in the absence of IFN-γ signaling, is associated with the presence of lesions in the more rostral regions of the brainstem, cerebellum, and brain in contrast to classical flaccid paralysis where lesions are more concentrated in the caudal regions of the spinal cord [8], [12]. These studies highlight the dominant role of IFN-γ in imposing classical EAE over atypical EAE and indicate that a lack of IFN-γ ‘allows’ atypical EAE. Thus, atypical EAE in IFN-γ replete rodents may be due to immunization strategies that cause strongly biased Th2 polarization and consequent down-regulation of IFN-γ in the context of chronic immune stimulation against MOG.

Atypical EAE has also been observed in rats. Atypical EAE was reported in a limited proportion of DA rats immunized with IgV-MOG in IFA but not in CFA as indicated by the presence of ‘brain-stem’ demyelinating lesions and ataxia as the sole clinical symptom [41]. Sporadic occurrence of atypical EAE was similarly noted in some LEW.1AV1 rats immunized with rat IgV-MOG in CFA as was evident as an uncontrolled body rotation [42]. Atypical EAE likewise occurred in LEW.N rats and several other rat strains bearing the RT1^n^ B/D MHC class II alleles upon immunization with rat IgV-MOG in IFA as was noted by clinical signs such as forelimb paralysis, severe balance disturbance, and continuous rotatory spinning [43]. Aside from one study [42], an atypical phenotype in rats was associated with the use of IFA rather than CFA.

### The IFA adjuvant, the antigen, and atypical EAE

The association of IFA-based booster immunizations with atypical or non-classical EAE (Figures 1, 2, 3, 7) dovetails with the association of the IFA adjuvant with Th2 or Th17 dominant responses and a paucity of IFN-γ production. The heat-killed *Mycobacterium* component of CFA is known to prime Th1 responses and IFN-γ production. In accordance, immunization of various rat strains with rat IgV-MOG in CFA favored induction of classical rather than atypical EAE [41], [43]. Conversely, IFA may elicit progressively less IFN-γ production over time and a more pronounced reactivity by alternative T cell subsets that may promote atypical EAE. This concept is in accordance with our study in that one or more boosters in IFA represented a key variable needed for induction of atypical EAE in Lewis rats (Figures 1–2).

Antigen may regulate the balance between classical and atypical EAE by an influence on APC preference. Although IgV-MOG in IFA may initially elicit a weak Th1 response in Lewis rats, thereby accounting for the initial self-limited bout of classical EAE, the emergence of a strong humoral immune response may shift the dominant APC subset from dendritic cells to IgV-MOG specific B cells. Clonal expansion of antigen-specific B cells exponentially increases the predominance of B cell APC because B cells use antigen-specific surface immunoglobulin to capture antigen for subsequent presentation. Emergence of IgV-MOG-specific B cells as a predominant APC subset may in turn polarize the immune response to alternative Th2 or Th17 responses and counter-regulate Th1 T cell-mediated IFN-γ production. In turn, lack of IFN-γ production may restrict access to various regions of the CNS and thereby favor atypical EAE. Thus, a globular antigen (IgV-MOG) that drives a vigorous polyclonal B cell response may downregulate IFN-γ production that enables predominance of the type and distribution of lesions that favor atypical EAE.

### Atypical EAE in a cynomolgus macaque

Three NHP models of EAE are prominent, including two in old world species, *Macaca fascicularis* (cynomolgus macaques) and *Macaca mulatta* (rhesus macaques), and one in a new world species *Callithrix jacchus* (marmosets) [19], [20]. Cynomolgus and rhesus macaques are closely related, and the evolutionary span between rhesus macaques and humans is approximately 35 million years. Even specific mechanisms of cognate T cell antigen recognition are cross-reactive between these primate species and humans [21], [44]–[48]. Marmosets are separated from humans by approximately 55 million years in evolutionary time. The Macaque species represent a primary choice for translational research due to the close genetic link to humans.

These three species of NHP were tested for susceptibility to EAE by repeated immunization with recombinant human MOG protein (1–125 aa extracellular domain) in IFA adjuvant [49]. In four cynomolgus macaques, 1–2 immunizations elicited a rapidly progressive disease that reached the humane endpoint within 3 days. Another cynomolgus NHP had a monophasic course of mild ataxic EAE with complete recovery but after a subsequent booster exhibited paresis that progressed within 2 days to a moribund state. CNS lesions in these NHP were often associated with neutrophil infiltration, necrosis, hemorrhage, and demyelination. In two rhesus macaques, a primary immunization and one boost caused a rapidly progressive disease that within two days of onset caused complete paraplegia or quadriplegia in association with large demyelinating plaques in brain including some hemorrhagic lesions primarily populated with neutrophilic granulocytes. Two additional rhesus macaques that received four immunizations however did not show clinical or histological EAE. Marmosets, like the cynomolgus and rhesus macaques, also exhibited a high incidence of an acute CNS demyelinating syndrome that rapidly progressed in 3–9 days to reach humane endpoints [49], [50]. These experiments were based on the use of MOG antigens in IFA but nonetheless resulted in a high incidence of an acute paralysis reminiscent of classical EAE and were in contrast to the mild monophasic disease course shown in Figure 7 of this study.

The differences in acute lethal EAE noted in [49] and the mild monophasic disease (Figure 7) may be related to the several considerations. First and most importantly, Figure 7 is based on a single NHP that may not be representative of a larger cohort. Second, we used a mix of rat and macaca IgV-MOG as opposed to human IgV-MOG. Differences in antigenic strength therefore may play a role in modulating disease, particularly in that ‘self’ determinants in the macaca IgV-MOG may engage regulatory T cells to limit disease progression and impose tolerance to MOG. Third, the folding, stability, and solubility of the IgV-MOG may be an important but unpredictable variable. Our IgV-MOG preparations were refolded from inclusion bodies and were soluble but would develop aggregates at high concentrations. Aggregated protein may have altered immunogenicity and may efficiently elicit strong T cell-mediated responses via presentation by myeloid APC. In contrast, soluble, natively-folded protein would more likely be captured and presented by MOG-specific B cells and therefore may more likely drive Th2 immunity and more complex disease syndromes of atypical EAE. Natively folded, glycosylated, highly soluble IgV-MOG monomers should be considered in future studies of NHP-EAE in rhesus monkeys.

The transient, reversible, myotonias observed in the macaque were reminiscent of an arousal-elicited episode of Uhthoff’s phenomena, which is an abrupt, fully-reversible worsening of neurological symptoms in MS of brief duration. Uhthoff’s attacks are often triggered by increased body temperature, physical stress, or psychological stress [51]. The macaque typically exhibited a transient episode of rigid paralysis in a large limb or involuntary balling of a hand or foot at the onset of an observation period, particularly the third or evening observation period. These deficits completely resolved in approximately 1–2 minutes. The sudden arousal associated with the onset of the daily observation periods appeared to elicit these episodes. Unlike classical EAE or hyperacute EAE, the presence of complex neurological episodes resembling the MS-associated Uhthoff’s syndrome in this atypical macaca model of EAE reinforces the association of this case with the complex neurological deficits of MS.

## Conclusion

The present study revealed a new model of atypical EAE in Lewis rats. This model represents an improvement over previously described rodent models of atypical EAE because the Lewis rat model reliably exhibited a high incidence of severe atypical disease. The study also provided a framework to study atypical/complex EAE in *Macaca fascicularis*. Although the scope of the NHP study was limited to one case, the disease had many interesting features. The disease was self-limited, was stable, did not progress to a humane endpoint, presented with complex neurological signs, and was associated with robust IgG response against conformational epitopes of macaca MOG. Notably, the NHP case was not associated with an ascending flaccid paralysis or other manifestations of classical EAE. Overall, the study showed that repeated booster immunization with refolded globular IgV-MOG in IFA is a key variable for induction of atypical or unusual forms of EAE. Development of reliable atypical models of EAE is an important goal for the field that will have important implications for identifying valid MS drug candidates.
