# Correction: The Extracellular Domain of Myelin Oligodendrocyte Glycoprotein Elicits Atypical Experimental Autoimmune Encephalomyelitis in Rat and *Macaque* Species

**DOI:** 10.1371/journal.pone.0117878

**Published:** 2015-02-06

**Authors:** The PLOS ONE Staff

Fig. 1 is an incorrect duplicate of Fig. 10. The publisher apologizes for the error. Please see the correct Fig. 1 here.

**Figure 1:**
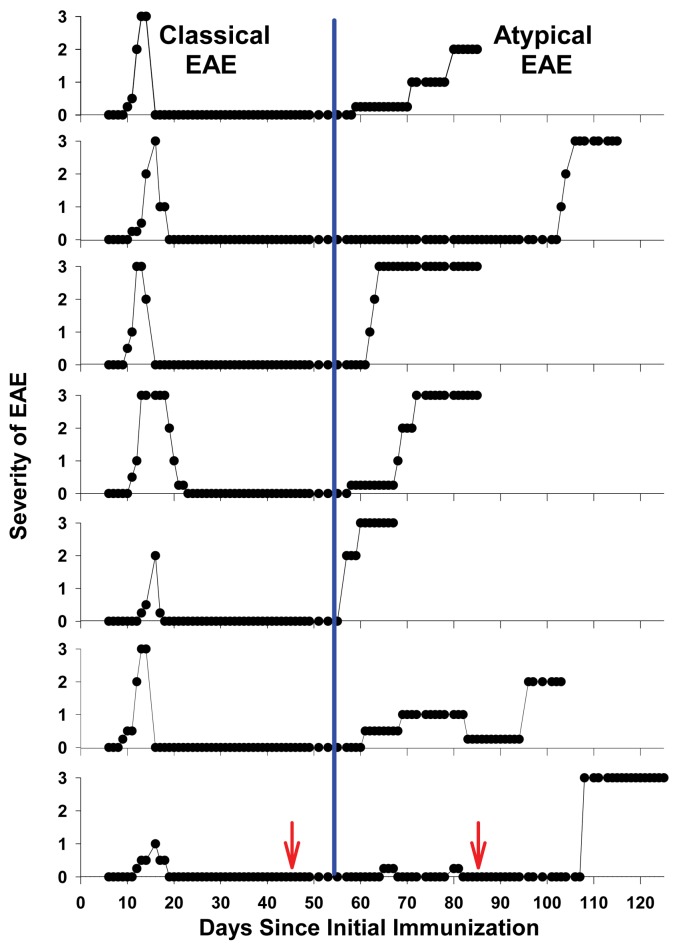
Booster immunizations with IgV-MOG in IFA caused a chronic course of atypical EAE. Seven Lewis rats were immunized with a mixture of 25 μg of GP69-88 and 50 μg of rat IgV-MOG in CFA on day 0. After resolution of a classical monophasic bout of EAE, the same rats were boosted with 200 μg of rat IgV-MOG in IFA on days 45 and 85. Rats were scored daily for clinical signs of EAE. After the IgV-MOG boosts in IFA, the incidence of chronic atypical EAE was 100% (7 of 7 rats). The red arrows (bottom panel) mark the dates of the boosts. Shown are the individual disease courses for the 7 rats. The blue line divides the clinical data into classical (left) and atypical (right) EAE courses.
